# Health system performance at the district level in Indonesia after decentralization

**DOI:** 10.1186/1472-698X-10-3

**Published:** 2010-03-05

**Authors:** Peter Heywood, Yoonjoung Choi

**Affiliations:** 1 Menzies Centre for Health Policy, University of Sydney, NSW, Australia; 2 Department of International Health, Johns Hopkins Bloomberg School of Public Health, Baltimore, MD, USA

## Abstract

**Background:**

Assessments over the last two decades have showed an overall low level of performance of the health system in Indonesia with wide variation between districts. The reasons advanced for these low levels of performance include the low level of public funding for health and the lack of discretion for health system managers at the district level. When, in 2001, Indonesia implemented a radical decentralization and significantly increased the central transfer of funds to district governments it was widely expected that the performance of the health system would improve. This paper assesses the extent to which the performance of the health system has improved since decentralization.

**Methods:**

We measured a set of indicators relevant to assessing changes in performance of the health system between two surveys in three areas: utilization of maternal antenatal and delivery care; immunization coverage; and contraceptive source and use. We also measured respondents' demographic characteristics and their living circumstances. These measurements were made in population-based surveys in 10 districts in 2002-03 and repeated in 2007 in the same 10 districts using the same instruments and sampling methods.

**Results:**

The dominant providers of maternal and child health in these 10 districts are in the private sector. There was a significant decrease in birth deliveries at home, and a corresponding increase in deliveries in health facilities in 5 of the 10 districts, largely due to increased use of private facilities with little change in the already low use of public facilities. Overall, there was no improvement in vaccination of mothers and their children. Of those using modern contraceptive methods, the majority obtained them from the private sector in all districts.

**Conclusions:**

There has been little improvement in the performance of the health system since decentralization occurred in 2001 even though there have also been significant increases in public funding for health. In fact, the decentralization has been limited in extent and structural problems make management of the system as a whole difficult. At the national level there has been no real attempt to envision the health system that Indonesia will need for the next 20 to 30 years or how the substantial public subsidy to this lightly regulated private system could be used in creative ways to stimulate innovation, mitigate market failures, improve equity and quality, and to enhance the performance of the system as a whole.

## Background

The Indonesian health system of today is the result of the many changes over the last 60 years. In the 1950s health facilities, which had a markedly curative orientation, consisted mainly of private and public hospitals, treatment clinics, most of which were public and concentrated on treatment of adults, and maternal and infant health clinics, also mostly public. In 1951 a new health system based on integration of preventive and curative medicine was introduced in the city of Bandung. The strategy under this new system, eventually known as the Bandung Plan, was to distribute health facilities and providers to the people by building and staffing a network of public health facilities throughout the country. The central, and eventually iconic, element of this new system was the health center at the sub-district level; it was complemented by a hospital at the district level. These facilities were staffed through obligatory government service for all new graduates in medicine, nursing and midwifery [1]. By the mid-1990s there were more than 7,000 health centers with an average population per center of less than 30,000, more than 20,000 health sub-centers and, starting in the mid-1980s, a program to locate midwives in villages. These facilities were the peripheral service delivery elements of a highly centralized, hierarchical system in which essentially no decisions of consequence were left to the district and facility level staff [2].

Assessments over the last two decades, however, have showed low performance of the system and have concluded that the low level of public funding for health, which was less then 0.5% of GDP between 1985 and 1999, was an important reason [3]. In addition, others argued that the hierarchical and authoritarian nature of government in Indonesia meant that local authorities were unable to address local problems, the expected nature of which varied greatly in such a geographically and culturally diverse country.

Despite these funding shortcomings Indonesia has made significant advances over the last 40 years in selected health indicators of a population such as infant mortality and life expectancy [4]. However, there has been less progress on other important health indicators such as maternal mortality [5] and immunization [6]. The health system shows few signs of responding to the changing disease patterns arising from the demographic and epidemiological transitions. In addition, there is wide variation between districts and regions in health status [7].

Assessments of health system performance at the national level have only limited value [8]. Much more useful information for policymakers could come from district level assessments of health system performance [9], particularly in a country as diverse as Indonesia. One approach to within-country analysis is to measure efficiency (outputs per quantity of input) at the district level and identify those districts and regions which are more efficient at producing the desired health system outcomes [10]. The Indonesian government has, in fact, carried out such an analysis using data from the end of the 1990s, immediately before decentralization. Not surprisingly, the results showed wide variation in district level health system performance [6]. The World Bank has recently updated this analysis using data from 2005 and 2006 [7] and again demonstrated wide variations in system efficiency - variations in health outcomes at the same level of inputs as well as variations in the level of inputs at the same outcomes. The further analysis also showed that district level health spending per capita is not related to critical health outcomes such as immunization coverage and skilled birth attendance, even after controlling for the size of the district economy and household expenditure [7], consistent with findings from other developing countries [11,12].

In 2001 Indonesia implemented a radical fiscal, political and administrative decentralization [13] under which the responsibility for delivery of services, including health, was transferred to the districts. This decentralization, together with the disappointing performance of the health system, raised expectations that the performance of the public sector would improve, not least because such a move had been widely advanced as a means of doing so [14,15]. Despite the weak relationship between health spending and health outcomes in Indonesia and elsewhere [16], expectations increased even further when, in the years following the formality of decentralization in 2001, there was a significant increase in public expenditure on health with real public expenditure more than doubling between 2000 and 2006 [3].

Eight years on, the question is whether the performance of the health system has improved since decentralization and, if so, whether any improvement is attributable to decentralization of health service delivery. To examine this, one would ideally conduct a district-randomized controlled trial, assessing performance changes before and after the experimental treatment (i.e., decentralization of health service delivery) between experiment and comparison districts. However, in reality decentralization is usually not a randomized, controlled trial in which its evaluation is amenable to the classical experimental approach. Such was also the case in Indonesia where decentralization was part of a broader process of political and economic reform following the downfall of the authoritarian regime of President Suharto. Decentralization, which occurred as part of widespread political change, involved transfer of responsibility for health service delivery from the central Ministry of Health to the district governments. At the same time, some fiscal, administrative and political authority was also transferred. This transfer occurred right across the country at the same time - there was no possibility of a control group and an experimental group. And given the turmoil at the time, baseline measurements were not a priority activity. Consequently, any attempt to assess change in performance after decentralization must take advantage of assessments made for other purposes around 2001 and then make comparable measurements at a later time. Even so, attribution of any change detected to 'decentralization' is not possible as there is no control group for comparison. Further, because decentralization is an ongoing process, especially in the first years after it occurs, attribution is also dependent on assessing the extent to which the decentralization actually occurred.

For the purposes of this assessment at the district level, health service statistics routinely reported by facility staff are not adequate for at least three reasons. First, routine statistics for some health system outcomes (e.g. immunization) have been shown to be misleading, in many cases providing estimates that are significantly above those levels shown in high quality population based surveys [17]. Second, the routine service statistics in Indonesia do not include the contribution made by the private sector. And third, since decentralization took place in Indonesia the functioning of an already weak health information system has likely become more unreliable, at least in some districts.

This study examines changes in performance of the health system since decentralization was introduced, using population based surveys conducted in 2002-03 and 20007 in 10 districts. The work reported here is part of a project to understand what is happening at the district level in the Indonesian health sector. It includes a basic enumeration of the human resources and the health facilities in which they work and deliver services as well as estimation of the financial resources available to health through the public purse at the district level. Our aim, in a sample of 15 districts in Java, is to: (i) enumerate the stock of health facilities (public and private) in the health sector in 2006; (ii) enumerate the stock of human resources (public and private) in the health sector in 2006 trained to provide care and treatment for illness - in Indonesia this means doctors, nurses and midwives; (iii) estimate the funds (public and private) spent on health care in the course of 2006; and (iv) assess whether the performance of the health system has improved. The current paper reports on health system performance. Other results of the project are reported in separate papers - those for health personnel in [1], health facilities in [18], and public funding of health in [2].

## Methods

### Data

Data come from a special part of the Indonesian Demographic and Health Surveys (IDHS) 2002-03 and IDHS 2007. IDHS was first conducted in Indonesia in 1987 and subsequently in 1991, 1994, 1997, 2002-03 and 2007. IDHS, as a part of DHS now conducted in more than 75 countries, is a nationally representative cross-sectional household survey and interviews all ever-married women between 15 to 49 years of age living in sampled households. IDHS collect individual demographic information and household characteristics for all sampled women. In addition, among women who had at least one live birth during the 5-year period before the survey, information on prenatal and delivery care is collected. For all children alive who are 5 years of age or younger, the survey collects information on preventive and curative child health care utilization. Interviews are conducted in both Bahasa Indonesia and participants' local language.

Typical IDHS sample design allows estimates of various indicators at the provincial level. In IDHS 2002-03, however, the sample size was increased in 10 districts to provide district level estimates for monitoring and evaluation of the Government of Indonesia/World Bank Safe Motherhood Project. The project was implemented in East Java Province and Central Java Province between 1997 and 2004, and the 10 districts were selected purposively by the project to illustrate the range of settings in which the project was implemented. They are Cilacap, Rembang, Jepara, Pemalang, and Brebes in Central Java and Trenggalek, Jombang, Ngawi, Sampang and Pamekasan in East Java. All 10 districts have low fiscal revenue per capita and low Gross Development Product per capita, based on the World Bank classification [3]. Table 1 presents further information on the selected districts. In each of the 10 districts, the typical IDHS sample design was expanded to allow for estimates at the district level for all of the main variables in the standard DHS. Detailed information on district-level sampling is given in [4]. In IDHS 2007, the district-level survey was repeated in the same 10 districts. In 2002-03 the data was collected by BPS-Statistics Indonesia as part of the national IDHS with funding from the Safe Motherhood Project. The dataset set for that survey was obtained from BPS-Statistics Indonesia. In 2007 the survey in the 10 districts was also carried out as part of the national IDHS, this time at the request of Australian Health Policy Institute at the University of Sydney and with funding from the Ford Foundation. In both cases the survey was approved as part of the national survey implemented by BPS-Statistics Indonesia, the national statistics organization of the Government of Indonesia.

**Table 1:** Basic information about the 10 districts included in this study.

Province	District	Population (in year 2006)	No. Sub-districts
Central Java	Brebes	1727708	17
	Cilacap	1717273	24
	Jepara	1078037	14
	Pemalang	1341422	14
	Rembang	591786	14
East Java	Jombang	1203716	21
	Ngawi	857449	19
	Pamekasan	782917	13
	Sampang	801541	14
	Trenggalek	682328	14

Source: Various District Health Office Annual Reports for 2007.

The surveys were conducted between October, 2002 and January, 2003 and between July and September of 2007. In total, 4555 and 3324 eligible women were sampled in the 10 districts in 2002-03 and 2007, respectively (Table 2). Of these, 98.6 percent and 97.3 percent completed interview in 2002-03 and 2007, respectively. The lowest district response rate was 90.2 percent in Trenggalek; all other response rates were above 95 percent. Table 2 presents response rate by survey and district. Both district surveys were carried out by BPS-Statistics Indonesia as a part of standard IDHS 2002-03 and 2007, using the same questionnaire and survey implementation procedures [4].

**Table 2:** Response rates (%) for eligible households and women 15-49 years old by district for surveys in 2003 and 2007.

Year	District	Number of eligible households, unweighted	Number of eligible households with complete interview, unweighted	Eligible household response rate (%)	Number of eligible women, unweighted	Number of eligible women with complete interview, unweighted	Eligible women response rate (%)
2003	CJ: Cilacap	499	479	96.0	356	354	99.4
	CJ: Rembang	500	486	97.2	420	410	97.6
	CJ: Jepara	500	476	95.2	454	454	100.0
	CJ: Pemalang	500	487	97.4	497	492	99.0
	CJ: Brebes	500	484	96.8	475	458	96.4
	EJ: Trenggalek	500	496	99.2	544	543	99.8
	EJ: Jombang	499	499	100.0	391	380	97.2
	EJ: Ngawi	499	491	98.4	387	380	98.2
	EJ: Sampang	500	485	97.0	464	460	99.1
	EJ: Pamekasan	500	495	99.0	567	560	98.8
	**Total**	**4,997**	**4,878**	**97.6**	**4,555**	**4,491**	**98.6**
2007	CJ: Cilacap	400	395	98.8	307	304	99.0
	CJ: Rembang	400	398	99.5	352	351	99.7
	CJ: Jepara	400	394	98.5	362	357	98.6
	CJ: Pemalang	400	391	97.8	347	345	99.4
	CJ: Brebes	400	392	98.0	314	304	96.8
	EJ: Trenggalek	399	387	97.0	337	304	90.2
	EJ: Jombang	400	393	98.3	303	298	98.3
	EJ: Ngawi	400	393	98.3	287	279	97.2
	EJ: Sampang	400	393	98.3	331	316	95.5
	EJ: Pamekasan	400	391	97.8	384	375	97.7
	**Total**	**4000**	**3927**	**98.2**	**3324**	**3233**	**97.3**

(CJ: Central Java; EJ: East Java).

### Measurements

We use a set of indicators relevant to assessing changes in performance of the health system between the two surveys in three areas: utilization of antenatal and delivery care; immunization coverage; and contraceptive method use. Individual indicators and their detailed definition are shown in Table 3. The indicators used are drawn from those measured in the Demographic and Health Surveys and, thus, place special emphasis on maternal and child health and contraception. Whilst it is acknowledged that these do not represent the whole health system they do cover areas to which the health system in all countries claim to give special emphasis, allocate a high proportion of funds, and to which the international community urges particular attention through the Millennium Development Goals. We also measured respondents' demographic characteristics and their living circumstances.

**Table 3:** Indicators of health system performance and background characteristics and health system performance indicators

**Maternal background characteristics, among women who had live birth in 5 years preceding the survey**
Age less than 30 years
Completed primary school or higher
Do not read newspaper of magazine at all
Listen to radio less than once a week/not at all
Watch TV almost every day
Living in a household with dirt floor
Living in a household where cooking fuel is firewood/straw/bush
Living in a household with protected drinking water sources
Living in a household with sanitary toilet

Antenatal care utilization, among women who were pregnant in 5 years preceding the survey*
Antenatal care provided by health professional
Antenatal care received at private medical sector
TT immunization 2 times or more
Received prenatal iron tablets

**Delivery care utilization, among women who had live birth in 5 years preceding the survey †**
Delivery at home
Delivery in a facility
Delivery in a private medical sector

**Immunization, among children 12-23 months old**
Mother has a vaccination card, seen by the interviewer
Received all: BCG, 3 DPT, 3 Polio, & Measles, based on either the vaccination card or maternal report.

**Contraception use, among all ever-married women 15-49 years, currently not pregnant**
Doing something to avoid pregnancy
Using modern contraceptive methods
Modern methods obtained at private medical sector

* Last pregnancy was analyzed if a woman had multiple pregnancies during the period

^† ^Last live birth was analyzed if a woman had multiple pregnancies during the period

### Statistical analyses

The primary aim of analyses was to assess whether the performance of the health system in these 10 districts has changed in the period between two surveys. We estimated proportions of eligible population meeting the definition of each indicator with a 95% confidence interval by survey and district, and compared changes between 2002-03 and 2007 in each district. All analyses were adjusted for sampling weights. STATA 9.0 statistical software (Stata Corporation, College Station, TX, USA) was used for all analyses.

### Study limitations

The study design has a number of limitations. Given the way in which decentralization occurred it was not possible to use an experimental design with experimental and control groups; consequently clear attribution of any changes that may have occurred to decentralization is not possible. In addition, the 10 districts were not randomly selected - they were selected purposively during the implementation of the Safe Motherhood project to represent the range of circumstances of the districts included in the project. As such, the districts are broadly representative (but not necessarily in a statistical sense) of the districts on Java (where 60% of the population live) but not of the districts in other parts of Indonesia. Further, the sample size at the district level may not provide sufficient statistical power to detect all real differences in individual indicators that may have emerged in the period between the two surveys. This is more so for some indicators (e.g. vaccination) in which the numbers in both surveys are relatively small. For many indicators the differences between the two surveys are small and differences unlikely to be detected even with considerably larger sample sizes. Even so, as is evident in the results below, there is a consistency in the results across the three sets of indicators (antenatal and delivery care, child immunization coverage, and contraceptive method use) that give a strong indication that the effects as a whole are coherent. In separate studies we have assessed the situation in human resources for health and public funding in 2007 and these data assist in interpreting the interpreting the results of the current study. However, we do not have similar personnel and financing data for 2002-03.

## Results

### Changes in maternal characteristics and living situation

Background characteristics of the mothers who had a birth during the five years before each survey are shown in Table 4 and Additional File 1. Between 2002/03 and 2007 there was no significant change in the proportion of mothers less than 30 years of age, except in Sampang. The proportion with primary school education or more improved in all districts and the increase was statistically significant in Cilacap, Trenggalek and Ngawi. The proportion not reading newspapers or magazines did not change significantly, except in Cilacap. The proportion watching TV almost everyday increased in five districts. However, the proportion listening to radio less than once increased in four districts.

**Table 4:** Changes in proportions of women with selected background demographic and household characteristics in 10 districts between 2002/03 and 2007

Province and District	Mothers < 30 years			Completed primary school or higher			Do not read newspaper or magazine			Listen to radio less than once a week					
	2002	2007		2002	2007		2002	2007		2002	2007				
**Central Java**															
Cilacap	**0.45**	**0.41**		**0.73**	**0.94**	S	**0.78**	**0.49**	S	**0.65**	**0.76**				
Rembang	**0.51**	**0.5**		**0.87**	**0.94**		**0.55**	**0.62**		**0.68**	**0.75**				
Jepara	**0.63**	**0.49**		**0.87**	**0.91**		**0.72**	**0.62**		**0.66**	**0.71**				
Pemalang	**0.6**	**0.57**		**0.69**	**0.87**		**0.57**	**0.5**		**0.36**	**0.86**	^S^			
Brebes	**0.55**	**0.56**		**0.56**	**0.72**		**0.58**	**0.72**		**0.5**	**0.82**	^S^			
**East Java**															
Trenggalek	**0.58**	**0.6**		**0.91**	**0.99**	S	**0.6**	**0.44**		**0.48**	**0.47**				
Jombang	**0.49**	**0.47**		**0.94**	**0.96**		**0.58**	**0.45**		**0.31**	**0.59**	^S^			
Ngawi	**0.58**	**0.65**		**0.8**	**0.93**	S	**0.65**	**0.54**		**0.48**	**0.81**	^S^			
Sampang	**0.57**	**0.8**	S	**0.36**	**0.61**		**0.83**	**0.69**		**0.84**	**0.73**				
Pamekasan	**0.59**	**0.62**		**0.61**	**0.83**		**0.69**	**0.63**		**0.77**	**0.79**				
															
	**Watch TV almost every day**			**Live in house with dirt floor**			**Use cooking fuel wood/straw/bush**			**Live in house with protected drinking water source**			**Live in house with sanitary toilet**		
	**2002**	**2007**		**2002**	**2007**		**2002**	**2007**		**2002**	**2007**		**2002**	**2007**	
**Central Java**															
Cilacap	**0.5**	**0.8**	S	**0.25**	**0.16**		**0.58**	**0.5**		**0.69**	**0.65**		**0.37**	**0.63**	
Rembang	**0.68**	**0.8**		**0.51**	**0.36**		**0.51**	**0.49**		**0.54**	**0.52**		**0.34**	**0.51**	
Jepara	**0.66**	**0.85**	S	**0.35**	**0.3**		**0.48**	**0.67**		**0.74**	**0.65**		**0.25**	**0.43**	S
Pemalang	**0.66**	**0.71**		**0.43**	**0.2**		**0.74**	**0.61**		**0.66**	**0.62**		**0.13**	**0.42**	
Brebes	**0.64**	**0.71**		**0.35**	**0.22**		**0.67**	**0.57**		**0.63**	**0.6**		**0.29**	**0.46**	
**East Java**															
Trenggalek	**0.57**	**0.83**	S	**0.29**	**0.15**		**0.79**	**0.74**		**0.5**	**0.33**		**0.2**	**0.51**	
Jombang	**0.81**	**0.84**		**0.18**	**0.16**		**0.28**	**0.45**		**0.72**	**0.91**		**0.49**	**0.56**	
Ngawi	**0.7**	**0.9**	S	**0.67**	**0.33**		**0.82**	**0.48**		**0.64**	**0.66**		**0.33**	**0.53**	
Sampang	**0.31**	**0.65**	S	**0.57**	**0.44**		**0.72**	**0.72**		**0.34**	**0.67**		**0.15**	**0.3**	
Pamekasan	**0.58**	**0.63**		**0.32**	**0.21**		**0.6**	**0.54**		**0.77**	**0.88**		**0.24**	**0.37**	

Significant change based on 95% confidence interval. A more detailed version of this table showing 95% confidence intervals and numbers for each comparison is shown in Additional File 1.

There was no significant change in indicators of living conditions, including living in a house with a dirt floor, cooking with wood/fuel/bush, living in a house with a protected drinking water source. The proportion of women living in a house with sanitary toilet improved in all districts, but was significant only in Pemalang.

### Antenatal care and delivery

The proportion of mothers who received antenatal care from a health professional was high in all districts in 2002-03 (> 0.90 in 8 of the 10 districts) and there were no significant changes in 2007 (Table 5 and Additional File 2). Among those who received antenatal care, most women received it from a private sector provider (nurse, midwife, village midwife) across districts, and there were no significant differences between the two surveys.

**Table 5:** Changes in proportions of women receiving selected antenatal and delivery care in 10 districts between 2002/03 and 2007

Province and District	Antenatal care provided by a health professional			Antenatal care by private sector health professional, among those who visited any health professional			TT immunization two times or more in last pregnancy			Prenatal iron tables		
	2002	2007		2002	2007		2002	2007		2002	2007	
**Central Java**												
Cilacap	**0.97**	**0.96**		**0.82**	**0.83**		**0.78**	**0.57**	S	**0.94**	**0.9**	
Rembang	**0.98**	**0.96**		**0.61**	**0.72**		**0.62**	**0.49**		**0.87**	**0.88**	
Jepara	**0.96**	**0.97**		**0.94**	**0.9**		**0.53**	**0.61**		**0.92**	**0.87**	
Pemalang	**0.95**	**0.93**		**0.67**	**0.77**		**0.55**	**0.42**		**0.83**	**0.84**	
Brebes	**0.95**	**0.97**		**0.62**	**0.68**		**0.7**	**0.61**		**0.88**	**0.83**	
**East Java**												
Trenggalek	**0.95**	**0.99**		**0.7**	**0.76**		**0.74**	**0.31**	S	**0.91**	**0.87**	
Jombang	**0.96**	**1**		**0.91**	**0.93**		**0.56**	**0.6**		**0.94**	**0.97**	
Ngawi	**0.97**	**0.86**		**0.75**	**0.78**		**0.64**	**0.56**		**0.92**	**0.76**	S
Sampang	**0.77**	**0.86**		**0.46**	**0.57**		**0.12**	**0.45**	S	**0.65**	**0.71**	
Pamekasan	**0.89**	**0.96**		**0.67**	**0.62**		**0.34**	**0.17**	S	**0.76**	**0.68**	
												
	**Delivered at home**			**Delivered in a health facility**			**Delivered in a private health facility, among facility deliveries**					
	**2002**	**2007**		**2002**	**2007**		**2002**	**2007**				
**Central Java**												
Cilacap	**0.69**	**0.32**		**0.31**	**0.68**		**0.76**	**0.86**				
Rembang	**0.85**	**0.59**	S	**0.15**	**0.41**	S	**0.59**	**0.75**				
Jepara	**0.65**	**0.55**		**0.35**	**0.45**		**0.91**	**0.95**				
Pemalang	**0.79**	**0.68**		**0.21**	**0.32**		**0.7**	**0.77**				
Brebes	**0.76**	**0.52**		**0.24**	**0.48**		**0.89**	**0.86**				
**East Java**												
Trenggalek	**0.57**	**0.43**		**0.43**	**0.57**		**0.73**	**0.57**				
Jombang	**0.33**	**0.07**	S	**0.67**	**0.93**	S	**0.83**	**0.84**				
Ngawi	**0.48**	**0.12**	S	**0.52**	**0.88**	S	**0.73**	**0.84**				
Sampang	**0.88**	**0.55**	S	**0.12**	**0.45**	S	**0.62**	**0.82**				
Pamekasan	**0.79**	**0.36**	S	**0.21**	**0.64**	S	**0.82**	**0.84**				

Significant change based on 95% confidence interval. A more detailed version of this table showing 95% confidence intervals and numbers for each comparison is shown in Additional File 2.

However, there was wide variation across districts in receipt of prenatal tetanus toxoid immunization and prenatal iron tablets (Table 5 and Additional File 2). The proportion receiving tetanus toxoid (TT) twice or more ranged from 0.12 to 0.78 in 2002-03, and the proportion significantly decreased in three districts (Cilacap, Trenggalek, Pamekasan) but increased in Sampang, off a very low base of 0.12. The proportion receiving prenatal iron tablets was relatively lower in Sampang and Pamekasan in 2002-03, compared to over 0.80 in the rest of the districts; there was no clear pattern of change between the two surveys, except a significant decrease in Ngawi (from 0.92 in 2002-03 to 0.76 in 2007).

Indicators of delivery care improved between 2002-03 and 2007 (Table 5 and Additional File 2). The proportion of mothers who delivered at home decreased in all districts, and the decrease was significant in 5 districts (4 in East Java and 1 in Central Java). There was a significant increase in the proportion of deliveries in a health facility in the 5 districts that showed a significant decrease in home deliveries. Among deliveries at facilities, the majority was in private facilities (most with a nurse/midwife/village midwife, results not shown); there was no significant change over time in the type of facility utilized for delivery.

### Vaccination

Among mothers of children 12-23 months of age, the proportion able to show the interviewer a vaccination card was less than 0.60 in all districts in 2002-03 - as low as 0.09 in Sampang and 0.27 in Pamekasan (Table 6 and Additional File 3). There was no significant trend in all districts. Consistent with these results, the proportion of children between 12-23 months of age who were fully vaccinated was less than 0.50 in all districts in 2002-03. The proportion increased in all districts in 2007, but was significant in only Trenggalek and Ngawi. We expanded our analyses to children 6-59 months of age with similar results (results not shown).

**Table 6:** Changes in indicators of childhood immunization and use of contraceptive methods in 10 districts between 2002/03 and 2007 (proportion)

	Mothers who have vaccination card, seen by interviewer		Received all vaccinations: BCG, DPT, polio, measles			Doing something to avoid pregnancy			Using modern contraceptive methods, among those doing something to avoid pregnancy			Obtained modern contraceptive methods at private sector, among those using the method		
	2002	2007	2002	2007		2002	2007		2002	2007		2002	2007	
Central Java														
Cilacap	**0.53**	**0.84**	**0.50**	**0.84**		**0.63**	**0.60**		**0.92**	**0.92**		**0.59**	**0.73**	S
Rembang	**0.51**	**0.56**	**0.36**	**0.42**		**0.69**	**0.68**		**0.98**	**0.97**		**0.51**	**0.69**	
Jepara	**0.48**	**0.53**	**0.21**	**0.37**		**0.60**	**0.66**		**0.96**	**0.97**		**0.86**	**0.88**	
Pemalang	**0.35**	**0.50**	**0.23**	**0.20**		**0.52**	**0.59**		**0.98**	**0.99**		**0.75**	**0.87**	
Brebes	**0.58**	**0.59**	**0.29**	**0.47**		**0.63**	**0.65**		**0.98**	**0.97**		**0.74**	**0.75**	
East Java														
Trenggalek	**0.47**	**0.88**	**0.22**	**0.84**	S	**0.59**	**0.72**		**0.91**	**0.94**		**0.56**	**0.67**	
Jombang	**0.43**	**0.42**	**0.28**	**0.30**		**0.71**	**0.69**		**0.96**	**0.98**		**0.68**	**0.79**	
Ngawi	**0.48**	**0.83**	**0.37**	**0.83**	S	**0.69**	**0.73**		**0.98**	**0.97**		**0.56**	**0.75**	S
Sampang	**0.09**	**0.09**	**0.09**	**0.07**		**0.45**	**0.58**		**0.89**	**0.97**	S	**0.61**	**0.60**	
Pamekasan	**0.27**	**0.30**	**0.14**	**0.29**		**0.38**	**0.62**	S	**1.00**	**0.91**	S	**0.69**	**0.63**	

S - Significant change based on 95% confidence interval. A more detailed version of this table showing 95% confidence intervals and numbers for each comparison is shown in Additional File 3.

### Contraception

Among all ever-married women between 15 to 49 years of age, the proportion doing something to avoid pregnancy ranged from 0.38 in Pamekasan in 2002-03 to 0.73 in Ngawi in 2007; there was no clear pattern of changes between surveys across the 10 districts (Table 6 and Additional File 3). Of those taking steps to avoid pregnancy, about 90% or higher were using modern methods across districts and surveys. Of those using modern contraceptive methods, the majority obtained them from the private sector in all districts. The proportion obtaining modern methods from the private sector increased in all districts, except Trenggalek, though the increase was significant in only two, Ngawi and Cilacap.

## Discussion

We assessed changes in utilization of maternal and child health services, as an indicator of health system performance, in 10 districts in East and Central Java, Indonesia, following decentralization in 2001. Our findings suggest a picture of a health system struggling to maintain, let alone improve, what is, at best, a mediocre overall performance [6,7]. Even though the public system was originally set up with a strong maternal and child health emphasis, performance of the maternal and child health services between 2002-03 and 2007 has shown little, if any, improvement in these 10 districts. Tetanus toxoid coverage actually decreased in 3 districts, there was no improvement in coverage of iron tablets, and there was no improvement in the immunization of children even though the base was already low. At the same time there was a dramatic strengthening of the trend to deliver in a health facility; the remarkable aspect of this trend is that mothers continued to opt for private sector facilities in preference to public sector facilities which showed negligible improvement in their share of deliveries.

Moreover, these results indicate that the dominant provider of maternal and child health services is the private sector which now accounts for the majority of antenatal care, deliveries in health facilities and family planning services, including provision of modern contraceptives. And this is occurring in a system originally set up to provide a special emphasis on women and children and the integration of prevention and treatment through the publicly funded health centers; a publicly funded system that has received, in addition to that provided by the government, major support from all the big aid donors over the last 25 years at the district-level.

Of course, some districts do better than others, but the picture is of variable efficiency (outputs in relation to inputs) around a low average level. This is consistent with the government's own analyses showing wide variation in efficiency of resource utilization between districts. As would be expected in such a system, the productivity of the central institution, the health center, is also variable around a low average [P Heywood, NP Harahap. Health center productivity in West Java Province, Indonesia. Unpublished.]. As the public system struggles, the population continues to move to the private sector providers for their services.

Fifty years ago when the health system was designed Indonesia was a very different place. The disease pattern was different, dominated by communicable diseases and high levels of maternal, infant and child mortality. Poverty levels were high and population mobility low, limited by a meager transport infrastructure. There were few health providers and facilities and very limited access to services. The health system was designed to overcome these problems. Emphasis was on communicable diseases, women and children, and on prevention as well as treatment. Reflecting the consensus of the period (later confirmed at the Alma Ata meeting in 1978), these services were to be funded and delivered through the public sector. Given the situation at the time, access was to be improved by establishing a public health system based on a health center in each sub-district and a hospital in each district.

A number of assessments have indicated sub-optimal performance of the health system. Some have expressed the hope that decentralization and increased funding would improve performance. Using results from 10 districts in Java, the work reported here, which covers the period of decentralization and increased public funding, indicates that the performance of the system has not improved between 2002 and 2007. This naturally begs the questions - why? Trying to answer this question means that first we need to stand back and look at the health system, how we got to this point and how it operates now. There are seven important aspects of the system which help address the question.

### This is a private system

The most striking aspect is that this is basically a private health system - it was already like that before the 2002-03 survey, as a result there was little further increase in private sector share by 2007. Although it did not start out that way, two-thirds of the financing and more than half of the services are now private [7]. For example, reproductive health services are now delivered predominantly by private providers. This is despite that fact that the original emphasis of the public system, as it was designed in the 1960s and 1970s, was the funding and delivery of maternal and child health services. The current situation evolved slowly. When the government conscripted and assigned new graduates (doctors, nurses and midwives) to public health centers and hospitals it also gave them the right to private practice to supplement their low government salaries. The result is that almost all doctors, nurses and midwives have a private practice. The strength of the private practices evolved over time to the point where now, in a typical district in Java, the "...distribution of facilities and providers means that within each sub-district there is a range of facility types (multi-provider, solo-provider); a range of provider types in the facilities (doctors, nurses and midwives); a range of facility locations, some close to, even in the village, others in the sub-district headquarters. Some facilities are public, others private; some are free, at others there is a charge for the service. This distribution allows consumers to exercise some choice of facility and/or provider. In exercising this choice, many consumers choose the solo-provider facilities even though the public facilities at the health center, sub-center and district hospital are nominally free and they have to pay out-of-pocket at the private practice of the doctor, nurse and midwife. The lower fees of the nurse and midwife mean that they are often the preferred choice of the poor." [18] For many, the private sector is the sector of choice for outpatient care, in many cases because the private sector providers and facilities are much more consumer oriented and convenient than those in the public sector.

The transition to a predominantly private system has been ignored by the government which still, as evidenced by its health strategy statements, acts as if the public sector is the main player [19]. In fact, over the last two years the central government has moved to hire large numbers of new permanent civil servants in the health sector, again acting as if the public sector is the main provider of health services. It does this even as there is increasing recognition of the low level of productivity in public sector facilities like the health center. In the absence of increased patients, adding more staff to public sector facilities will further reduce productivity (and efficiency) per provider.

### .......with a heavy public subsidy

Recognizing that the health system is now a predominantly private system does not, of itself, improve sector performance, but it is a necessary first step. The main reason is that this is a private system with a very substantial public subsidy, principally in the form of the salaries of the providers who work for the government. This subsidy potentially provides the government with an important policy lever for the health sector. However, apart from the government's reluctance to use the funds in this way, the government has severely limited its capacity to maneuver by recently converting contract staff to permanent civil servants [1]. The flexibility of the district government to change staffing levels in response to changing circumstances is now severely limited by the permanent nature of these appointments as well as the continuing reluctance of the government to address the need for civil service reform.

### The health system is fractured making it difficult to manage the system as a whole

Even as the government continues to act as if it is the only game in town it also turns away from managing the system as a whole. Whilst this is partly a manifestation of the central ministry's reluctance to acknowledge that it is no longer the biggest game in town when it comes to either funding or delivery of services, there are other reasons why management of the sector is difficult. In essence the system at the district level is fractured along a number of different lines [2]. The public/private split is one fracture line. Equally important is the split between the district and the central government. The district, which is where about half of the public funds for health are actually spent, no longer feels beholden to the center in the way it had to be in the Suharto era. Nevertheless, the possibility of increased central funding for negotiated purposes means that districts can be pulled back into line, if necessary. But perhaps the most serious fracture line making management of the health system at the district level difficult is that between the district hospital and the district health office/health centers. These two sets of organizations, funded by the district, as well as the central government are, nevertheless, managed and funded separately by the districts, and the central ministry relates to them separately. For these reasons there are few districts in which there is a coherent view of the health system and where it is going; and nothing has emerged from the central ministry to indicate that they are doing this either. Finally, there is a fracture line around the funds over which the district does or does not have discretion. Overall, the districts have discretion over less than a third of the public funds for health spent in the district; but the proportion over which discretion can be exercised is much higher in hospitals and much less in the district health office/health center. Consequently health centers, between which there is considerable variation in settings and circumstances, are much less able to respond to variation in settings than are hospitals, where the range of settings is usually much less. These various fracture lines make management of the health system as a whole more difficult. The result is that districts fail to acknowledge the important role of the private sector and provide incentives for private providers to contribute to an improved sector performance. This is even more critical now that an increasing proportion of doctors and midwives work only in their private practice and have no direct contact with the government [1]. This is a marked contrast with the situation 20 years ago when all doctors, nurses and midwives worked as civil servants and engaged in private practice after office hours. Now in some districts as much as 30% of doctors work on their own account and have no formal contact with the government. Consequently the government knows very little about them and what happens in their practice. An important manifestation of this unwillingness to include the private sector is the markedly inadequate staff at the district level to oversee and monitor quality of private sector services.

As the ability to manage a fractured system is impaired, the other major casualty is that accountability is lost. Eventually, no one is held accountable for the performance of the sector - the district blames the center and the central ministries (and their ministers) are not accountable to district populations.

### The decentralization that wasn't

The underlying notion of decentralization is an expansion of choice at the local level [20], something that was very much expected when decentralization was introduced in 2001. In the Indonesian health sector this expansion of choice or discretion over the use of funds at the district level was brief, to the extent that it occurred at all. From the moment decentralization occurred, the central bureaucracy moved to re-establish control over use of public funds by the districts. Thus, despite high hopes and strong rhetoric, the extent to which decentralization actually occurred is limited [2]. The best example of the limited extent to which decentralization has occurred relates to sector human resources. Before decentralization, the districts had essentially no control over hiring and firing of permanent civil servants but had created some flexibility in their wages costs and skills mix by hiring a significant number of staff on contract to the local health facility in addition to the complement of permanent national civil servants. After decentralization the central government continued to control permanent civil servants and, in the last two years, has moved to convert the contract staff to permanent civil servants. As with existing civil servants, the salaries of these new civil servants have first call on the 'unallocated' funds transferred from the central government. That is, an increasing proportion of public funds is consumed by salaries for permanent civil servants further reducing the discretion of districts over their public funds. In effect, much of the increased funds are not available for operational purposes. The decision by the central government to convert almost all contract staff to permanent civil servants means that districts have lost almost all flexibility with respect to their most important resource category.

### Incentives for innovation are very limited

Despite high hopes, the system has not really changed under decentralization. The basic structure of the district health services is as it was before; likewise the basic staffing and skills mix are essentially unchanged and inadequate to the task of determining why performance is low and what is needed to improve it at the district-level. Whilst the central government provides full payment for the cost of civil servants there is, in fact, a disincentive for districts to innovate [3] - why worry about the cost of staff when the central government pays the bill anyway? Apart from, the low levels of system performance and efficiency, the need for a new direction is given added emphasis by the change in disease patterns over the last 20 years from one dominated by communicable diseases to one in which non-communicable diseases are now foremost. Like many such complex systems, the health system in Indonesia is highly path dependent - decisions made 30 and 40 years ago about the structure and functioning of the health system continue to influence and constrain the direction in the future. To the extent that the central government continues to reinforce the current personnel system and restrict the fiscal and human resource choices of districts, in effect to maintain and reinforce the old system, the innovation so much needed for improvement in health system performance will continue to be discouraged.

### Low quality services

Another important factor potentially explaining low utilization is low quality of health services in both the public and private sectors. The best study of service quality in Indonesia assessed the extent to which various types of providers operating in different parts of the health system had knowledge of clinical guidelines on antenatal care, child curative care and adult curative care. Overall, the knowledge was low. Doctors had the highest levels of knowledge, private nurses - the largest single group of health care providers [1] - the lowest, and private midwives in between the two [21]. Improving the quality of care provided by all groups, especially nurses, where the low tariff makes them the provider of choice for many of the poor, may be one of the most important avenues for improving quality of outpatient care, especially for those with low incomes. To date, attempts to improve their skills are opposed on the grounds that their private practice is illegal. The system does not monitor quality of care and there are no incentives to improve.

### Low capacity at the district level

The analytic and planning capacity of staff at all levels (district, province, center, civil society) is limited. Few senior members of the health sector at the district level are exercising real leadership and articulating a vision of what the sector needs to do to respond to the current and future health status and health sector issues. In the absence of leadership and vision bureaucrats keep on trying to implement the old system a little bit more, rather than look ahead to what is needed to respond to the health problems of today and take Indonesia into the future.

The structural issues in the sector (failure to recognize the importance of the private sector and the way in which the fractures are impeding sector change and development), the failure of decentralization, and the extent to which current government policies stifle innovation at the district level mean that it is difficult to improve performance. True, there were high hopes that decentralization would stimulate changes and innovation at the district level as local administrations used the newfound discretion over resources to tackle local problems in ways appropriate to the local situation. But the discretion has been continually reduced. After almost a decade of decentralization there are very few examples of innovative approaches to local health problems and the results reported here indicate that the performance of district health system has not improved. Without innovation this will remain an inefficient, low productivity, low quality, low performance system unable to address the health problems of the 21^st ^century.

## Conclusion

In 10 districts in East and Central Java, Indonesia, there was little improvement in utilization of maternal and child health services, one of the key indicators of health system performance. Government studies show that at the beginning of this decade there was wide variation between districts in efficiency of health resource use and that most district systems operated at sub-optimal levels. Even though there have been significant increases in public funds for health, recent studies show that not only has little changed, but also that there is no relationship between public spending on health at the district level and health system outputs. During this period there has also been a failure of leadership, political as well as bureaucratic, in the health sector. District and provincial politicians routinely resort to populist notions of free health care as a vote buying strategy; few of these politicians or their senior bureaucratic advisors are looking beyond the next election to determine what is needed to improve the performance of the health system and the health status of their populations. At the national level there has been no real attempt to envision the health system that Indonesia will need for the next 20 to 30 years or how the substantial public subsidy to this lightly regulated private system could be used in creative ways to stimulate innovation, mitigate market failures, improve equity and quality, and to enhance the performance of the system as a whole - instead, the emphasis is on continuing to implement the system of the past and regaining central control of the health system. All of these political and bureaucratic acts discourage the innovation and energy needed to build a new health system appropriate to the Indonesian health problems of today and, more importantly, tomorrow.

## Competing interests

The authors declare that they have no competing interests.

## Authors' contributions

PH conceived the study and drafted the manuscript. YC analyzed the data, assisted with interpretation of results and revision of the manuscript.

## Pre-publication history

The pre-publication history for this paper can be accessed here:

http://www.biomedcentral.com/1472-698X/10/3/prepub

## Supplementary Material

Additional file 1 **Maternal characteristics and living situation - estimated proportion, upper and lower limits of 95% confidence interval and un-weighted and weighted N, by district**. The file contains estimated proportions, together with upper and lower limits of 95% confidence interval and weighted and un-weighted N, of women having certain maternal characteristics for each of the 10 districts included in the study. Click here for file

Additional file 2 **Additional Table 2 - Antenatal care and delivery variables - estimated proportion, upper and lower limits of 95% confidence interval and un-weighted and weighted N, by district**. The file contains estimated proportions, together with upper and lower limits of 95% confidence interval and weighted and un-weighted N, for antenatal care and delivery variables for each of the 10 districts included in the study. Click here for file

Additional file 3 **Vaccination, childhood illness and contraception variables - estimated proportion, upper and lower limits of 95% confidence interval and un-weighted and weighted N, by district**. The file contains estimated proportions, together with upper and lower limits of 95% confidence interval and weighted and un-weighted N, for variables related to vaccination, childhood illness and contraception, for each of the 10 districts included in the study. Click here for file
