# Supplementary material for: Health system performance at the district level in Indonesia after decentralization
Source: BMC Int Health Hum Rights. 2010 Mar 5;10:3. doi: 10.1186/1472-698X-10-3 (PMC2839983; doi:10.1186/1472-698X-10-3)
Supplement: Additional file 1 — Maternal characteristics and living situation - estimated proportion, upper and lower limits of 95% confidence interval and un-weighted and weighted N, by district. The file contains estimated proportions, together with upper and lower limits of 95% confidence interval and weighted and un-weighted N, of women having certain maternal characteristics for each of the 10 districts included in the study. [file 1472-698X-10-3-S1.PDF]

Additional File 1. Maternal characteristics and living situation - estimated proportion, upper and lower limits of 95% confidence interval and un-weighted and weighted N, by district.

| Indicator | **2003** | | | | | **2007** | | | | | |
|---|---|---|---|---|---|---|---|---|---|---|---|
| | Proportion | LL | UL | Un-weighted N | weighted N | Proportion | LL | UL | N, unweighted | N, weighted | |
| Age less than 30 years | | | | | | | | | | | |
| CJ: Cilacap | **0.45** | 0.35 | 0.54 | 159 | 264 | **0.41** | 0.29 | 0.53 | 111 | 120 | |
| CJ: Rembang | **0.51** | 0.42 | 0.59 | 138 | 76 | **0.50** | 0.42 | 0.57 | 115 | 123 | |
| CJ: Jepara | **0.63** | 0.54 | 0.72 | 183 | 196 | **0.49** | 0.40 | 0.58 | 161 | 161 | |
| CJ: Pemalang | **0.60** | 0.52 | 0.68 | 202 | 221 | **0.57** | 0.47 | 0.67 | 121 | 119 | |
| CJ: Brebes | **0.55** | 0.47 | 0.63 | 214 | 369 | **0.56** | 0.48 | 0.64 | 138 | 134 | |
| EJ: Trenggalek | **0.58** | 0.46 | 0.69 | 122 | 83 | **0.60** | 0.44 | 0.75 | 84 | 81 | |
| EJ: Jombang | **0.49** | 0.40 | 0.57 | 134 | 136 | **0.47** | 0.37 | 0.58 | 111 | 111 | |
| EJ: Ngawi | **0.58** | 0.51 | 0.65 | 131 | 124 | **0.65** | 0.58 | 0.72 | 84 | 93 | |
| EJ: Sampang | **0.57** | 0.49 | 0.64 | 179 | 143 | **0.80** | 0.68 | 0.92 | 140 | 119 | * |
| EJ: Pamekasan | **0.59** | 0.54 | 0.65 | 183 | 120 | **0.62** | 0.44 | 0.81 | 150 | 168 | |
| Completed primary school or higher | | | | | | | | | | | |
| CJ: Cilacap | **0.73** | 0.66 | 0.80 | 159 | 264 | **0.94** | 0.89 | 0.99 | 111 | 120 | * |
| CJ: Rembang | **0.87** | 0.81 | 0.94 | 138 | 76 | **0.94** | 0.88 | 0.99 | 115 | 123 | |
| CJ: Jepara | **0.87** | 0.82 | 0.91 | 183 | 196 | **0.91** | 0.85 | 0.96 | 161 | 161 | |
| CJ: Pemalang | **0.69** | 0.58 | 0.81 | 202 | 221 | **0.87** | 0.78 | 0.96 | 121 | 119 | |
| CJ: Brebes | **0.56** | 0.44 | 0.68 | 214 | 369 | **0.72** | 0.60 | 0.83 | 138 | 134 | |
| EJ: Trenggalek | **0.91** | 0.86 | 0.95 | 122 | 83 | **0.99** | 0.97 | 1.00† | 84 | 81 | * |
| EJ: Jombang | **0.94** | 0.89 | 0.99 | 134 | 136 | **0.96** | 0.92 | 0.99 | 111 | 111 | |
| EJ: Ngawi | **0.80** | 0.72 | 0.87 | 131 | 124 | **0.93** | 0.87 | 1.00 | 84 | 93 | * |
| EJ: Sampang | **0.36** | 0.24 | 0.49 | 179 | 143 | **0.61** | 0.41 | 0.80 | 140 | 119 | |
| EJ: Pamekasan | **0.61** | 0.47 | 0.76 | 183 | 120 | **0.83** | 0.73 | 0.94 | 150 | 168 | |
| Do not read newspaper of magazine at all | | | | | | | | | | | |
| CJ: Cilacap | **0.78** | 0.69 | 0.88 | 159 | 264 | **0.49** | 0.31 | 0.67 | 111 | 120 | * |
| CJ: Rembang | **0.55** | 0.43 | 0.68 | 138 | 76 | **0.62** | 0.44 | 0.79 | 115 | 123 | |
| CJ: Jepara | **0.72** | 0.62 | 0.82 | 183 | 196 | **0.62** | 0.52 | 0.72 | 161 | 161 | |
| CJ: Pemalang | **0.57** | 0.48 | 0.65 | 202 | 221 | **0.50** | 0.39 | 0.60 | 121 | 119 | |
| CJ: Brebes | **0.58** | 0.48 | 0.67 | 214 | 369 | **0.72** | 0.59 | 0.85 | 138 | 134 | |
| EJ: Trenggalek | **0.60** | 0.51 | 0.69 | 122 | 83 | **0.44** | 0.24 | 0.63 | 84 | 81 | |
| EJ: Jombang | **0.58** | 0.46 | 0.69 | 134 | 136 | **0.45** | 0.35 | 0.56 | 111 | 111 | |
| EJ: Ngawi | **0.65** | 0.55 | 0.75 | 131 | 124 | **0.54** | 0.34 | 0.73 | 84 | 93 | |
| EJ: Sampang | **0.83** | 0.74 | 0.92 | 179 | 143 | **0.69** | 0.54 | 0.83 | 140 | 119 | |
| EJ: Pamekasan | **0.69** | 0.59 | 0.80 | 183 | 120 | **0.63** | 0.49 | 0.76 | 150 | 168 | |
| Listen to radio less than once a week/not at all | | | | | | | | | | | |
| CJ: Cilacap | **0.65** | 0.56 | 0.74 | 159 | 264 | **0.76** | 0.70 | 0.83 | 111 | 120 | |
| CJ: Rembang | **0.68** | 0.62 | 0.73 | 138 | 76 | **0.75** | 0.65 | 0.86 | 115 | 123 | |

| | | | | | | | | | | | |
|---|---|---|---|---|---|---|---|---|---|---|---|
| CJ: Jepara | **0.66** | 0.59 | 0.72 | 183 | 196 | **0.71** | 0.62 | 0.79 | 161 | 161 | |
| CJ: Pemalang | **0.36** | 0.29 | 0.43 | 202 | 221 | **0.86** | 0.81 | 0.91 | 121 | 119 | * |
| CJ: Brebes | **0.50** | 0.43 | 0.58 | 214 | 369 | **0.82** | 0.71 | 0.92 | 138 | 134 | * |
| EJ: Trenggalek | **0.48** | 0.38 | 0.58 | 122 | 83 | **0.47** | 0.35 | 0.59 | 84 | 81 | |
| EJ: Jombang | **0.31** | 0.19 | 0.42 | 134 | 136 | **0.59** | 0.46 | 0.71 | 111 | 111 | * |
| EJ: Ngawi | **0.48** | 0.39 | 0.58 | 131 | 124 | **0.81** | 0.73 | 0.89 | 84 | 93 | * |
| EJ: Sampang | **0.84** | 0.78 | 0.90 | 179 | 143 | **0.73** | 0.63 | 0.83 | 140 | 119 | |
| EJ: Pamekasan | **0.77** | 0.70 | 0.84 | 183 | 120 | **0.79** | 0.69 | 0.88 | 150 | 168 | |
| Watch TV almost every day | | | | | | | | | | | |
| CJ: Cilacap | **0.50** | 0.36 | 0.65 | 159 | 264 | **0.80** | 0.68 | 0.91 | 111 | 120 | * |
| CJ: Rembang | **0.68** | 0.57 | 0.80 | 138 | 76 | **0.80** | 0.73 | 0.87 | 115 | 123 | |
| CJ: Jepara | **0.66** | 0.58 | 0.73 | 183 | 196 | **0.85** | 0.78 | 0.92 | 161 | 161 | * |
| CJ: Pemalang | **0.66** | 0.54 | 0.79 | 202 | 221 | **0.71** | 0.58 | 0.83 | 121 | 119 | |
| CJ: Brebes | **0.64** | 0.57 | 0.71 | 214 | 369 | **0.71** | 0.60 | 0.83 | 138 | 134 | |
| EJ: Trenggalek | **0.57** | 0.42 | 0.72 | 122 | 83 | **0.83** | 0.72 | 0.94 | 84 | 81 | * |
| EJ: Jombang | **0.81** | 0.74 | 0.89 | 134 | 136 | **0.84** | 0.79 | 0.89 | 111 | 111 | |
| EJ: Ngawi | **0.70** | 0.60 | 0.80 | 131 | 124 | **0.90** | 0.81 | 0.99 | 84 | 93 | * |
| EJ: Sampang | **0.31** | 0.19 | 0.43 | 179 | 143 | **0.65** | 0.43 | 0.87 | 140 | 119 | * |
| EJ: Pamekasan | **0.58** | 0.47 | 0.69 | 183 | 120 | **0.63** | 0.47 | 0.80 | 150 | 168 | |
| Living in a household with dirt/earth floor | | | | | | | | | | | |
| CJ: Cilacap | **0.25** | 0.15 | 0.36 | 159 | 264 | **0.16** | 0.03 | 0.28 | 111 | 120 | |
| CJ: Rembang | **0.51** | 0.34 | 0.69 | 138 | 76 | **0.36** | 0.19 | 0.53 | 115 | 123 | |
| CJ: Jepara | **0.35** | 0.25 | 0.45 | 183 | 196 | **0.30** | 0.18 | 0.41 | 161 | 161 | |
| CJ: Pemalang | **0.43** | 0.27 | 0.58 | 202 | 221 | **0.20** | 0.12 | 0.28 | 121 | 119 | |
| CJ: Brebes | **0.35** | 0.24 | 0.47 | 214 | 369 | **0.22** | 0.08 | 0.35 | 138 | 134 | |
| EJ: Trenggalek | **0.29** | 0.18 | 0.39 | 122 | 83 | **0.15** | 0.05 | 0.25 | 84 | 81 | |
| EJ: Jombang | **0.18** | 0.01 | 0.34 | 134 | 136 | **0.16** | 0.05 | 0.27 | 111 | 111 | |
| EJ: Ngawi | **0.67** | 0.54 | 0.80 | 131 | 124 | **0.33** | 0.07 | 0.58 | 84 | 93 | |
| EJ: Sampang | **0.57** | 0.43 | 0.71 | 179 | 143 | **0.44** | 0.22 | 0.65 | 140 | 119 | |
| EJ: Pamekasan | **0.32** | 0.19 | 0.44 | 183 | 120 | **0.21** | 0.07 | 0.35 | 150 | 168 | |
| Living in a household where cooking fuel is firewood/straw/bush | | | | | | | | | | | |
| CJ: Cilacap | **0.58** | 0.41 | 0.76 | 159 | 264 | **0.50** | 0.25 | 0.75 | 111 | 120 | |
| CJ: Rembang | **0.51** | 0.32 | 0.69 | 138 | 76 | **0.49** | 0.29 | 0.70 | 115 | 123 | |
| CJ: Jepara | **0.48** | 0.36 | 0.60 | 183 | 196 | **0.67** | 0.53 | 0.81 | 161 | 161 | |
| CJ: Pemalang | **0.74** | 0.60 | 0.87 | 202 | 221 | **0.61** | 0.42 | 0.80 | 121 | 119 | |
| CJ: Brebes | **0.67** | 0.56 | 0.77 | 214 | 369 | **0.57** | 0.45 | 0.69 | 138 | 134 | |
| EJ: Trenggalek | **0.79** | 0.71 | 0.88 | 122 | 83 | **0.74** | 0.60 | 0.88 | 84 | 81 | |
| EJ: Jombang | **0.28** | 0.19 | 0.38 | 134 | 136 | **0.45** | 0.29 | 0.60 | 111 | 111 | |
| EJ: Ngawi | **0.82** | 0.71 | 0.92 | 131 | 124 | **0.48** | 0.22 | 0.74 | 84 | 93 | |
| EJ: Sampang | **0.72** | 0.54 | 0.90 | 179 | 143 | **0.72** | 0.37 | 1.00† | 140 | 119 | |

| | | | | | | | | | | | |
|---|---|---|---|---|---|---|---|---|---|---|---|
| EJ: Pamekasan | **0.60** | 0.46 | 0.74 | 183 | 120 | **0.54** | 0.25 | 0.83 | 150 | 168 | |
| Living in a household with protected drinking water sources | | | | | | | | | | | |
| CJ: Cilacap | **0.69** | 0.52 | 0.85 | 159 | 264 | **0.65** | 0.52 | 0.78 | 111 | 120 | |
| CJ: Rembang | **0.54** | 0.37 | 0.71 | 138 | 76 | **0.52** | 0.28 | 0.75 | 115 | 123 | |
| CJ: Jepara | **0.74** | 0.64 | 0.83 | 183 | 196 | **0.65** | 0.56 | 0.74 | 161 | 161 | |
| CJ: Pemalang | **0.66** | 0.52 | 0.80 | 202 | 221 | **0.62** | 0.47 | 0.77 | 121 | 119 | |
| CJ: Brebes | **0.63** | 0.44 | 0.83 | 214 | 369 | **0.60** | 0.38 | 0.82 | 138 | 134 | |
| EJ: Trenggalek | **0.50** | 0.35 | 0.65 | 122 | 83 | **0.33** | 0.16 | 0.51 | 84 | 81 | |
| EJ: Jombang | **0.72** | 0.51 | 0.94 | 134 | 136 | **0.91** | 0.86 | 0.96 | 111 | 111 | |
| EJ: Ngawi | **0.64** | 0.41 | 0.87 | 131 | 124 | **0.66** | 0.56 | 0.76 | 84 | 93 | |
| EJ: Sampang | **0.34** | 0.15 | 0.52 | 179 | 143 | **0.67** | 0.44 | 0.90 | 140 | 119 | |
| EJ: Pamekasan | **0.77** | 0.68 | 0.86 | 183 | 120 | **0.88** | 0.81 | 0.96 | 150 | 168 | |
| Living in a household with sanitary toilet | | | | | | | | | | | |
| CJ: Cilacap | **0.37** | 0.22 | 0.52 | 159 | 264 | **0.63** | 0.38 | 0.87 | 111 | 120 | |
| CJ: Rembang | **0.34** | 0.19 | 0.48 | 138 | 76 | **0.51** | 0.36 | 0.66 | 115 | 123 | |
| CJ: Jepara | **0.25** | 0.12 | 0.37 | 183 | 196 | **0.43** | 0.28 | 0.57 | 161 | 161 | |
| CJ: Pemalang | **0.13** | 0.07 | 0.19 | 202 | 221 | **0.42** | 0.27 | 0.57 | 121 | 119 | * |
| CJ: Brebes | **0.29** | 0.18 | 0.41 | 214 | 369 | **0.46** | 0.33 | 0.58 | 138 | 134 | |
| EJ: Trenggalek | **0.20** | 0.06 | 0.35 | 122 | 83 | **0.51** | 0.29 | 0.74 | 84 | 81 | |
| EJ: Jombang | **0.49** | 0.35 | 0.63 | 134 | 136 | **0.56** | 0.43 | 0.69 | 111 | 111 | |
| EJ: Ngawi | **0.33** | 0.13 | 0.53 | 131 | 124 | **0.53** | 0.43 | 0.64 | 84 | 93 | |
| EJ: Sampang | **0.15** | 0.05 | 0.26 | 179 | 143 | **0.30** | 0.00† | 0.64 | 140 | 119 | |
| EJ: Pamekasan | **0.24** | 0.11 | 0.38 | 183 | 120 | **0.37** | 0.27 | 0.46 | 150 | 168 | |

LL: Lower Limit, UL: Upper Limit, CJ: Central Java, EJ: East Java
* Significant difference base on 95% confidence intervals
