# Supplementary material for: Health system performance at the district level in Indonesia after decentralization
Source: BMC Int Health Hum Rights. 2010 Mar 5;10:3. doi: 10.1186/1472-698X-10-3 (PMC2839983; doi:10.1186/1472-698X-10-3)
Supplement: Additional file 2 — Additional Table 2 - Antenatal care and delivery variables - estimated proportion, upper and lower limits of 95% confidence interval and un-weighted and weighted N, by district. The file contains estimated proportions, together with upper and lower limits of 95% confidence interval and weighted and un-weighted N, for antenatal care and delivery variables for each of the 10 districts included in the study. [file 1472-698X-10-3-S2.PDF]

Additional File 2. Antenatal care and delivery variables - estimated proportion, upper and lower limits of 95% confidence interval and un-weighted and weighted N, by district.

	2003					2007					
	Proportion	LL	UL	Un-weighted N	Weighted N	Proportion	LL	UL	Un-weighted N	Weighted N	
Antenatal care provided by health professional											
CJ: Cilacap	**0.97**	0.95	0.99	159	264	**0.96**	0.92	1.00	111	120	
CJ: Rembang	**0.98**	0.95	1.00†	138	76	**0.96**	0.92	1.00	115	123	
CJ: Jepara	**0.96**	0.93	0.99	183	196	**0.97**	0.95	1.00	161	161	
CJ: Pemalang	**0.95**	0.90	1.00	202	221	**0.93**	0.89	0.97	121	119	
CJ: Brebes	**0.95**	0.92	0.98	214	369	**0.97**	0.94	1.00	138	134	
EJ: Trenggalek	**0.95**	0.90	1.00	122	83	**0.98**	0.95	1.00†	84	81	
EJ: Jombang	**0.96**	0.94	0.99	134	136	**0.99**	0.97	1.00†	111	111	
EJ: Ngawi	**0.97**	0.94	1.00†	131	124	**1.00**	.	.	84	93	
EJ: Sampang	**0.77**	0.65	0.88	179	143	**0.86**	0.77	0.96	140	119	
EJ: Pamekasan	**0.89**	0.81	0.97	183	120	**0.86**	0.78	0.93	150	168	
Antenatal care received at private medical sector											
CJ: Cilacap	**0.82**	0.73	0.91	153	256	**0.83**	0.76	0.90	108	115	
CJ: Rembang	**0.61**	0.48	0.73	137	75	**0.72**	0.58	0.85	114	120	
CJ: Jepara	**0.94**	0.88	0.99	176	189	**0.90**	0.85	0.96	157	157	
CJ: Pemalang	**0.67**	0.56	0.78	194	213	**0.77**	0.62	0.91	114	111	
CJ: Brebes	**0.62**	0.50	0.74	205	352	**0.68**	0.56	0.79	135	130	
EJ: Trenggalek	**0.70**	0.57	0.82	122	83	**0.76**	0.66	0.87	83	80	
EJ: Jombang	**0.91**	0.85	0.97	132	134	**0.93**	0.88	0.97	110	110	
EJ: Ngawi	**0.75**	0.60	0.91	128	122	**0.78**	0.63	0.93	84	93	
EJ: Sampang	**0.46**	0.31	0.61	161	129	**0.57**	0.41	0.74	133	115	
EJ: Pamekasan	**0.67**	0.55	0.79	177	117	**0.62**	0.42	0.81	143	160	
TT immunization 2 times or more											
CJ: Cilacap	**0.78**	0.70	0.87	159	264	**0.57**	0.50	0.64	111	120	*
CJ: Rembang	**0.62**	0.52	0.72	138	76	**0.49**	0.36	0.61	115	123	
CJ: Jepara	**0.53**	0.41	0.65	183	196	**0.61**	0.54	0.68	161	161	
CJ: Pemalang	**0.55**	0.45	0.65	202	221	**0.42**	0.31	0.54	121	119	
CJ: Brebes	**0.70**	0.62	0.79	214	369	**0.61**	0.46	0.75	138	134	
EJ: Trenggalek	**0.74**	0.63	0.86	122	83	**0.31**	0.19	0.44	84	81	*

EJ: Jombang	**0.56**	0.49	0.63	134	136	**0.60**	0.50	0.71	111	111	
EJ: Ngawi	**0.64**	0.58	0.71	131	124	**0.56**	0.38	0.74	84	93	
EJ: Sampang	**0.12**	0.05	0.18	179	143	**0.45**	0.24	0.66	140	119	*
EJ: Pamekasan	**0.34**	0.27	0.41	183	120	**0.17**	0.09	0.26	150	168	*
Received prenatal iron tablets											
CJ: Cilacap	**0.94**	0.90	0.98	159	264	**0.90**	0.85	0.95	111	120	
CJ: Rembang	**0.87**	0.80	0.93	138	76	**0.88**	0.85	0.92	115	123	
CJ: Jepara	**0.92**	0.87	0.96	183	196	**0.87**	0.83	0.92	161	161	
CJ: Pemalang	**0.83**	0.76	0.90	202	221	**0.84**	0.77	0.91	121	119	
CJ: Brebes	**0.88**	0.83	0.94	214	369	**0.83**	0.73	0.93	138	134	
EJ: Trenggalek	**0.91**	0.86	0.96	122	83	**0.87**	0.78	0.96	84	81	
EJ: Jombang	**0.94**	0.90	0.98	134	136	**0.97**	0.92	1.00†	111	111	
EJ: Ngawi	**0.92**	0.88	0.97	131	124	**0.76**	0.69	0.84	84	93	*
EJ: Sampang	**0.65**	0.55	0.76	179	143	**0.71**	0.52	0.91	140	119	
EJ: Pamekasan	**0.76**	0.62	0.89	183	120	**0.68**	0.59	0.77	150	168	
Delivery at home											
CJ: Cilacap	**0.69**	0.51	0.87	183	299	**0.32**	0.09	0.56	119	132	
CJ: Rembang	**0.85**	0.74	0.95	145	80	**0.59**	0.45	0.74	126	135	*
CJ: Jepara	**0.65**	0.50	0.80	204	218	**0.55**	0.38	0.72	176	176	
CJ: Pemalang	**0.79**	0.73	0.84	222	243	**0.68**	0.55	0.81	133	131	
CJ: Brebes	**0.76**	0.66	0.86	246	425	**0.52**	0.35	0.69	147	143	
EJ: Trenggalek	**0.57**	0.26	0.88	128	86	**0.43**	0.18	0.69	88	85	
EJ: Jombang	**0.33**	0.20	0.46	146	148	**0.07**	0.02	0.11	122	123	*
EJ: Ngawi	**0.48**	0.39	0.58	145	140	**0.12**	0.00†	0.25	89	99	*
EJ: Sampang	**0.88**	0.78	0.97	222	180	**0.55**	0.36	0.75	165	131	*
EJ: Pamekasan	**0.79**	0.68	0.89	195	128	**0.36**	0.19	0.53	166	180	*
Delivery in a facility											
CJ: Cilacap	**0.31**	0.13	0.49	183	299	**0.68**	0.44	0.91	119	132	
CJ: Rembang	**0.15**	0.05	0.26	145	80	**0.41**	0.26	0.55	126	135	*
CJ: Jepara	**0.35**	0.20	0.50	204	218	**0.45**	0.28	0.62	176	176	
CJ: Pemalang	**0.21**	0.16	0.27	222	243	**0.32**	0.19	0.45	133	131	
CJ: Brebes	**0.24**	0.14	0.34	246	425	**0.48**	0.31	0.65	147	143	
EJ: Trenggalek	**0.43**	0.12	0.74	128	86	**0.57**	0.31	0.82	88	85	

EJ: Jombang	**0.67**	0.54	0.80	146	148	**0.93**	0.89	0.98	122	123	*
EJ: Ngawi	**0.52**	0.42	0.61	145	140	**0.88**	0.75	1.00†	89	99	*
EJ: Sampang	**0.12**	0.03	0.22	222	180	**0.45**	0.25	0.64	165	131	*
EJ: Pamekasan	**0.21**	0.11	0.32	195	128	**0.64**	0.47	0.81	166	180	*
Delivery AT private medical sector											
CJ: Cilacap	**0.76**	0.50	1.00†	48	93	**0.86**	0.78	0.94	58	89	
CJ: Rembang	**0.59**	0.44	0.74	26	12	**0.75**	0.61	0.88	46	55	
CJ: Jepara	**0.91**	0.85	0.98	78	76	**0.95**	0.88	1.00†	80	79	
CJ: Pemalang	**0.70**	0.56	0.84	48	51	**0.77**	0.64	0.89	46	42	
CJ: Brebes	**0.89**	0.78	1.00†	65	101	**0.86**	0.77	0.94	60	69	
EJ: Trenggalek	**0.73**	0.65	0.80	36	37	**0.57**	0.39	0.76	46	48	
EJ: Jombang	**0.83**	0.76	0.89	108	99	**0.84**	0.77	0.92	114	115	
EJ: Ngawi	**0.73**	0.60	0.86	79	72	**0.84**	0.76	0.92	71	87	
EJ: Sampang	**0.62**	0.36	0.89	25	22	**0.82**	0.72	0.92	58	58	
EJ: Pamekasan	**0.82**	0.70	0.95	40	27	**0.84**	0.72	0.95	83	115	

LL: Lower Limit, UL: Upper Limit, CJ: Central Java, EJ: East Java
* Significant difference base on 95% confidence intervals
