# Supplementary material for: Health system performance at the district level in Indonesia after decentralization
Source: BMC Int Health Hum Rights. 2010 Mar 5;10:3. doi: 10.1186/1472-698X-10-3 (PMC2839983; doi:10.1186/1472-698X-10-3)
Supplement: Additional file 3 — Vaccination, childhood illness and contraception variables - estimated proportion, upper and lower limits of 95% confidence interval and un-weighted and weighted N, by district. The file contains estimated proportions, together with upper and lower limits of 95% confidence interval and weighted and un-weighted N, for variables related to vaccination, childhood illness and contraception, for each of the 10 districts included in the study. [file 1472-698X-10-3-S3.PDF]

Additional File 3. Vaccination, childhood illness and contraception variables - estimated proportion, upper and lower limits of 95% confidence interval and un-weighted and weighted N, by district.

	2003					2007					
	Proportion	LL	UL	Un-weighted N	Weighted N	Proportion	LL	UL	Un-weighted N	Weighted N	
Mother has a vaccination card, seen by the interview											
CJ: Cilacap	**0.53**	0.31	0.74	34	53	**0.84**	0.60	1.00†	19	16	
CJ: Rembang	**0.51**	0.34	0.68	25	14	**0.56**	0.34	0.78	29	30	
CJ: Jepara	**0.48**	0.36	0.60	44	49	**0.53**	0.30	0.77	35	35	
CJ: Pemalang	**0.35**	0.15	0.54	36	41	**0.50**	0.28	0.73	26	26	
CJ: Brebes	**0.58**	0.43	0.72	45	74	**0.59**	0.42	0.75	29	31	
EJ: Trenggalek	**0.47**	0.13	0.81	21	12	**0.88**	0.77	1.00†	19	13	
EJ: Jombang	**0.43**	0.16	0.70	28	29	**0.42**	0.13	0.72	23	23	
EJ: Ngawi	**0.48**	0.26	0.70	25	24	**0.83**	0.63	1.00†	18	15	
EJ: Sampang	**0.09**	0.00†	0.25	26	21	**0.09**	0.00†	0.21	33	28	
EJ: Pamekasan	**0.27**	0.09	0.46	37	25	**0.30**	0.17	0.43	39	51	
Received all: BCG, 3 DPT, 3 Polio, & Measles											
CJ: Cilacap	**0.50**	0.29	0.71	34	53	**0.84**	0.60	1.00†	19	16	
CJ: Rembang	**0.36**	0.10	0.62	25	14	**0.42**	0.23	0.60	29	30	
CJ: Jepara	**0.21**	0.07	0.36	44	49	**0.37**	0.16	0.58	35	35	
CJ: Pemalang	**0.23**	0.06	0.40	36	41	**0.20**	0.05	0.36	26	26	
CJ: Brebes	**0.29**	0.15	0.43	45	74	**0.47**	0.28	0.66	29	31	
EJ: Trenggalek	**0.22**	0.00†	0.44	21	12	**0.84**	0.69	0.99	19	13	*
EJ: Jombang	**0.28**	0.08	0.48	28	29	**0.30**	0.01	0.59	23	23	
EJ: Ngawi	**0.37**	0.18	0.56	25	24	**0.83**	0.63	1.00†	18	15	*
EJ: Sampang	**0.09**	0.00†	0.25	26	21	**0.07**	0.00†	0.16	33	28	
EJ: Pamekasan	**0.14**	0.01	0.26	37	25	**0.29**	0.15	0.42	39	51	
Doing something to avoid pregnancy											
CJ: Cilacap	**0.63**	0.56	0.70	329	538	**0.60**	0.54	0.67	282	281	
CJ: Rembang	**0.69**	0.63	0.75	390	221	**0.68**	0.62	0.73	341	340	
CJ: Jepara	**0.60**	0.54	0.66	435	440	**0.66**	0.61	0.71	338	338	
CJ: Pemalang	**0.52**	0.47	0.58	474	528	**0.59**	0.53	0.65	333	332	
CJ: Brebes	**0.63**	0.58	0.68	440	749	**0.65**	0.60	0.71	293	293	
EJ: Trenggalek	**0.59**	0.55	0.64	527	357	**0.72**	0.62	0.81	295	297	

EJ: Jombang	**0.71**	0.65	0.76	367	415	**0.69**	0.63	0.75	292	292	
EJ: Ngawi	**0.69**	0.63	0.76	369	338	**0.73**	0.64	0.82	264	269	
EJ: Sampang	**0.45**	0.35	0.54	435	343	**0.58**	0.51	0.65	301	291	
EJ: Pamekasan	**0.38**	0.32	0.45	540	362	**0.62**	0.53	0.72	362	359	*
Using modern contraceptive methods, among those using any method											
CJ: Cilacap	**0.92**	0.86	0.98	202	339	**0.92**	0.85	0.99	167	170	
CJ: Rembang	**0.98**	0.97	1.00†	271	152	**0.97**	0.95	0.99	232	231	
CJ: Jepara	**0.96**	0.94	0.99	253	265	**0.97**	0.96	0.99	223	223	
CJ: Pemalang	**0.98**	0.95	1.00†	247	277	**0.99**	0.97	1.00†	196	195	
CJ: Brebes	**0.98**	0.97	1.00†	279	471	**0.97**	0.94	1.00	192	191	
EJ: Trenggalek	**0.91**	0.86	0.95	296	212	**0.94**	0.90	0.97	200	213	
EJ: Jombang	**0.96**	0.93	0.98	245	293	**0.98**	0.96	1.00	204	202	
EJ: Ngawi	**0.98**	0.96	1.00†	254	234	**0.97**	0.94	0.99	199	196	
EJ: Sampang	**0.89**	0.85	0.92	185	153	**0.97**	0.93	1.00†	160	169	*
EJ: Pamekasan	**1.00**	0.99	1.00†	196	139	**0.91**	0.85	0.97	216	224	*
Modern methods obtained at private medical sector, among those using the method											
CJ: Cilacap	**0.59**	0.52	0.66	183	313	**0.73**	0.66	0.79	151	157	*
CJ: Rembang	**0.51**	0.42	0.60	267	150	**0.69**	0.57	0.80	226	223	
CJ: Jepara	**0.86**	0.78	0.94	243	255	**0.88**	0.83	0.94	217	217	
CJ: Pemalang	**0.75**	0.65	0.84	240	270	**0.87**	0.83	0.91	192	193	
CJ: Brebes	**0.74**	0.63	0.84	275	464	**0.75**	0.66	0.84	186	185	
EJ: Trenggalek	**0.56**	0.49	0.62	271	192	**0.67**	0.60	0.75	186	199	
EJ: Jombang	**0.68**	0.55	0.81	232	281	**0.79**	0.73	0.86	199	197	
EJ: Ngawi	**0.56**	0.47	0.65	251	230	**0.75**	0.69	0.81	193	189	*
EJ: Sampang	**0.61**	0.47	0.74	164	136	**0.60**	0.39	0.81	155	164	
EJ: Pamekasan	**0.69**	0.57	0.80	195	138	**0.63**	0.45	0.80	203	204	

LL: Lower Limit, UL: Upper Limit, CJ: Central Java, EJ: East Java
* Significant difference base on 95% confidence intervals
